# Molecular marker development and genetic diversity exploration in *Medicago polymorpha*

**DOI:** 10.7717/peerj.14698

**Published:** 2023-01-16

**Authors:** Hailong Ren, Zhenwu Wei, Bo Zhou, Xiang Chen, Qiang Gao, Zhibin Zhang

**Affiliations:** 1 College of Animal Science and Technology, Yangzhou University, Yangzhou, Jiangsu, China; 2 Guangzhou Academy of Agricultural Sciences, Guangzhou, Guangdong, China; 3 Hainan Sanya Test Center of Crop Breeding, Xinjiang Academy of Agricultural Sciences, Sanya, Hainan, China; 4 State Key Laboratory of Cotton Biology, Institute of Cotton Research, Chinese Academy of Agricultural Sciences, Anyang, Henan, China

**Keywords:** *Medicago polymorpha*, SLAF-seq, Molecular marker development

## Abstract

*Medicago polymorpha* L. (bur clover), an invasive plant species of the genus Medicago, has been traditionally used in China as an edible vegetable crop because of its high nutritive value. However, few molecular markers for *M. polymorpha* have been identified. Using the recently published high-quality reference genome of *M. polymorpha*, we performed a specific-locus amplified fragment sequencing (SLAF-seq) analysis of 10 *M. polymorpha* accessions to identify molecular markers and explore genetic diversity. A total of 52,237 high-quality single nucleotide polymorphisms (SNPs) were developed. These SNPs were mostly distributed on pseudochromosome 3, least distributed on pseudochromosome 7, and relatively evenly distributed on five other pseudochromosomes of *M. polymorpha*. Phenotypic analysis showed that there was a great difference in phenotypic traits among different *M. polymorpha* accessions. Moreover, clustering all *M. polymorpha* accessions based on their phenotypic traits revealed three groups. Both phylogenetic analysis and principal component analysis (PCA) of all *M. polymorpha* accessions based on SNP markers consistently indicated that all *M. polymorpha* accessions could be divided into three distinct groups (I, II, and III). Subsequent genetic diversity analysis for the 10 *M. polymorpha* accessions validated the effectiveness of the *M. polymorpha* germplasm molecular markers in China. Additionally, SSR mining analysis was also performed to identify polymorphic SSR motifs, which could provide valuable candidate markers for the further breeding of *M. polymorpha*. Since *M. polymorpha* genetics have not been actively studied, the molecular markers generated from our research will be useful for further research on *M. polymorpha* resource utilization and marker-assisted breeding.

## Introduction

The genus *Medicago* consists of approximately 87 species including the most important forage crop species *M. sativa* and the legume model species *M. truncatula* (Steele et al., 2010). *M. polymorpha*, a wild relative of the two species, is a high inbreeding annual medic considered to be the most polymorphic species in the genus (Del, Porqueddu & Ovalle, 2001; Paredes et al., 2002). It has been increasingly used for both animal feed and human consumption in recent years, as well as used successfully in green manure, pasture improvement, nitrogen-fixing (Denton et al., 2007; Nichols et al., 2007; Graziano et al., 2010), and as a nutritious vegetable consumed fresh or cooked in China. This species is native to the Mediterranean region spanning North Africa, Asia, and Europe (Lesins & Lesins, 1979), and is widely distributed as a common invasive plant in Africa, Northern Europe, Asia, America, and Australia. Its introduction into Chile from southern Spain or Portugal dates back 400–450 years (Del et al., 2000; Paredes et al., 2002), whereas it entered America and Australia in the mid to late 1800s (Haan & Barnes, 1998). Little is known about *M. polymorpha*’s history in China.

Over the past few decades, several studies have been carried out to uncover the molecular mechanism of morphological variation of *M. polymorpha*. Bullitta et al. (1994) analyzed 45 *M. polymorpha* accessions derived from a wide range of climatic and soil conditions in Sardinia using 12 biochemical markers and found that the degree of variation in isozyme sites was not high. Likewise, only one-third of the enzymatic markers appeared to be polymorphic when 16 Tunisian germplasm accessions were characterized by Hannachi, Boussaid & Marrakchi (1998), and low isozymatic diversity was found in 41 naturalized accessions from Chile when examined using 12 different isozyme systems (Paredes et al., 2000). Later, Paredes et al. (2002) obtained a similar result in a genetic divergence evaluation of 36 Chilean *M. polymorpha* accessions using 40 random amplification polymorphic DNA (RAPD) markers. Additionally, an analysis using five simple sequence repeat (SSR) markers indicated a low diversity level in eight accessions in China (Chu et al., 2010). Most recently, a high SSR polymorphism was reported in 14 *M. polymorpha* populations in Iran (Emami-Tabatabaei et al., 2021). In the aforementioned studies, only a limited number of molecular markers were used for their analyses. Most importantly, the chromosome-scale genome sequence of *M. polymorpha* using an integrated approach including Illumina, PacBio, and Hi-C technologies was successfully published in 2021 (Cui et al., 2021), which laid a foundation for research on genetic variations of *M. polymorpha*.

To develop large-scale markers for breeding and understand the genetic diversity of *M. polymorpha*, a specific-locus amplified fragment sequencing (SLAF-seq) of 10 *M. polymorpha* accessions from different regions in China was conducted in this study. It should be noted that there are very few available genetic resources for the species, so the development of a massive set of SNPs is of interest. The accessions used in this study were geographically representative and mainly collected from the main producing areas of *M. polymorpha*. Moreover, their phenotypic traits were notably different. These characteristics were helpful for the identification of genetic variations.

## Materials and Methods

### Sample collection and field experiment

In China, *M. polymorpha* mainly distributes in five major cultivation areas (Jiangsu province, Zhejiang province, Anhui province, Yunnan province, and Shanghai) with very limited distribution elsewhere. For SLAF-seq analysis, a total of 10 representative *M. polymorpha* individual accessions with distinct phenotypic characteristics were collected from these five different geographical regions. Among them, six accessions came from Jiangsu province (MP-2, MP-4, MP-5, MP-7, MP-11, and MP-13), and the other four accessions from the other four areas (MP-6 from Zhejiang, MP-8 from Shanghai, MP-3 from Anhui, and MP-9 from Yunnan). For plant growth form, MP-3 and MP-4 grown in lawn were prostrate, MP-13 grown in farmland was semi erect, and the other seven *M. polymorpha* accessions grown in farmland were erect (Table 1).

**Table 1:** Geographical origins and characteristics of the 10 *M. polymorpha* accessions evaluated in this study.

Accession	Habitat	Plant type	Source	Longitude/Latitude
MP-2	Farmland	Erect	Haimen, Jiangsu, China	31°87′N, 121°18′E
MP-3	Lawn	Prostrate	Hexian, Anhui, China	31°87′N, 118°33′E
MP-4	Lawn	Prostrate	Danyang, Jiangsu, China	32°01′N, 119°61′E
MP-5	Farmland	Erect	Yangzhong, Jiangsu, China	32°23′N, 119°80′E
MP-6	Farmland	Erect	Wenling, Zhejiang, China	28°37′N, 121°39′E
MP-7	Farmland	Erect	Jiangdu, Jiangsu, China	32°43′N, 119°57′E
MP-8	Farmland	Erect	Chongming, Shanghai, China	31°64′N, 121°57′E
MP-9	Farmland	Erect	Chuxiong, Yunnan, China	25°05′N, 101°53′E
MP-11	Farmland	Erect	Wuxi, Jiangsu, China	31°66′N, 120°24′E
MP-13	Farmland	Semi erect	Haian, Jiangsu, China	32°53′N, 120°47′E

**Note:**

Source: Collection place of *M. polymorpha* accession.

Each living accession was taken to the experimental fields of Yangzhou University, Jiangsu Province, China (119°40′E, 32°35′N) for ball planting. After the seeds of each accession were harvested in May, field experiments for phenotypic trait measurement were conducted in a randomized complete block design with three biological replications in October at Yangzhou University. There were two rows for each accession. The seeds of each genotype in each replication were sown in drill with a spacing of 0.5 m in rows and 1 m between two accessions. No fertilizer was used, but timely drainage between the plot ditch, artificial weeding, and timely irrigation were utilized.

### Phenotype measurement and data statistical analysis

At the beginning of each accession’s flowering time in April of the following year, six different agronomic traits (leaf length (cm), leaf width (cm), plant height (cm), branch number (*n*), stem diameter (cm) and stem-leaf ratio) were evaluated on 10 plants per replication. Branch number is the number of branches at the base (including the main stem), and plant height is the absolute height from the base of the main stem to the growing point of the main stem. Stem diameter refers to the diameter of the base of the main stem, measured with a vernier caliper. Analysis of agronomical data was carried out using analysis of variance (ANOVA) and the general linear model (GLM) of SAS (V.9.4). Cluster analysis was conducted using the Squared Euclidean Distance method with SPSS software (V.19.0).

### Extraction of genomic DNA and enzyme solution design

Young healthy leaves of each accession were collected, frozen in liquid nitrogen, and then stored at −80 °C before DNA extraction. Total high-quality genomic DNA was extracted using a minor modified CTAB method (Murray & Thompson, 1980). DNA concentration and quality were examined with an ND-1000 spectrophotometer (NanoDrop, Wilmington, DE, USA) and by electrophoresis in 1% agarose gels with a lambda DNA standard. DNA concentrations in all samples were adjusted to the same level for the construction of SLAF libraries. To obtain adequate SLAF tags, the restriction enzyme combination “RsaI + HaeIII” (NEB, Ipswich, MA, USA) was selected based on (a) lots of SLAF tags, (b) fewer restriction fragments with repeat sequences, (c) an even distribution across chromosomes, and (d) simulated fragments aligning uniquely to the reference genome.

### SLAF library construction and sequencing

SLAF-seq was performed with slight modifications as described previously (Sun et al., 2013). First, for the *in-silico* prediction of the number of markers produced by different enzymes, marker-discovery experiments were designed by analyzing the *M. truncatula* A17 (NCBI: taxid 3880; https://www.ncbi.nlm.nih.gov/Taxonomy/Browser/wwwtax.cgi?mode=info&id=3880) using prediction software independently developed by Biomarker Technologies Corporation (Beijing, China). Based on the result of the preliminary experiment, *Rsa*I and *Hae*III restriction enzymes were selected as the best combination. To further evaluate the accuracy of the ‘*Rsa*I + *Hae*III’ combination enzyme digestion experiment, *Arabidopsis thaliana* ecotype Columbia was used as the control for sequencing. Genomic DNA was then digested with restriction enzyme *Rsa*I (NEB, Ipswich, MA, USA), ligated to *Rsa*I adapter, and then digested by additional enzyme *Hae*III (NEB). Polymerase chain reactions (PCR) were carried out using the diluted restriction-ligation samples, dNTP, Taq DNA polymerase (NEB), and *Rsa* I-primer containing a barcode. According to the position of *Arabidopsis* reads in the genome and the length distribution of inserted fragments in *Arabidopsis thaliana* ecotype Columbia, DNA fragments of 314–414 bp for *M. polymorpha* were selected using the Blue Pippin automated DNA size selection system. We classified these as SLAF tags and then sequenced them using the Illumina HiSeq 2500 system (Illumina, Inc., San Diego, CA, USA) with 125 bp pair-end. The sequencing was performed by Biomarker Technologies Corporation (Beijing, China).

### Molecular marker development

SLAF marker identification and genotyping were performed as described by Sun et al. (2013). High-quality SLAF tags were obtained from 10 *M. polymorpha* genotypes. SLAFs with two to four tags were considered polymorphic SLAFs and potential markers. Polymorphic SLAF tags showed sequence polymorphisms between different accessions. Before mapping, low-quality reads with quality scores less than 30 were removed, and high-quality reads with terminal 5-bp and barcode sequences were discarded. All high-quality SLAF paired-end sample reads of the 10 accessions were then aligned to the newly assembled reference genomes of *M. polymorpha* ‘Huaiyang Jinhuacai’ (Cui et al., 2021; https://ngdc.cncb.ac.cn/gwh/Assembly/17540/show) using the Burrows-Wheeler alignment tool (BWA) software (Li & Durbin, 2009) with default parameters. Variants at each SLAF locus were called independently for each accession by GATK (v.4.0) (McKenna et al., 2010), and then the genotypes of all accessions (*n* = 10) were joined into a VCF file with the criteria of integrity >90%, minor allele frequency (MAF) >0.05, and max missing 0.5. The filtered high-quality SNPs were further used to reconstruct the phylogenetic tree with the maximum likelihood estimation method in MEGA-X (Kumar et al., 2018). The principal component analysis (PCA) of total individuals was performed using GCTA v1.93 (Yang et al., 2011). A three-dimensional (3D) point cloud was plotted using the “scatterplot3d” package in R 4.1.0 software (R Core Team, 2021) (https://CRAN.R-project.org/package=scatterplot3d).

### Genetic diversity analyses

The assessments of genetic diversity, including minor allele frequency (MAF), observed heterozygosity (Ho), expected heterozygosity (He), Nei diversity index (Nei), Shannon’s information index (I), and polymorphic information content (PIC), were calculated through Perl programming.

## Results

### Phenotypic characterization

There were significant differences in the phenotypic traits across the different *M. polymorpha* accessions. As shown in Table 2, the leaf length of *M. polymorpha* materials in this study varied from 1.38 to 2.40 cm, with a variation amplitude of 1.02 cm, and the leaf width varied from 1.45 to 2.23 cm, with an amplitude of 0.78 cm. Among all accessions, the leaf length and width of MP-6 from Wenling, Zhejiang province were the largest, and this material is also known as “Wenling big leaf” in the local area. We found that the germplasm characteristics of the MP-6 large leaves were not changed due to alloplanting. The leaf length and width of MP-4 from Danyang, Jiangsu province were the smallest, followed by MP-3 from Anhui, which retained more wild characteristics. The plant height of *M. polymorpha* accessions showed obvious differentiation. For example, the plant heights of MP-3 and MP-4 were only about 4 cm, and the lateral branches crept close to the ground because the main stem growth was significantly inhibited. However, other materials grew upright, ranging in height from 20 to 25 cm, except for MP-13. Moreover, the lateral branches of MP-3 and MP-4 were very developed and totaled up to 4, which was significantly more than that of the other materials. Stem diameter ranged from 1.71 to 2.08 cm, with an average of 1.89 cm. MP-9 from Chuxiong, Yunnan province had a stem-to-leaf ratio, of 1.33, indicating that it had the most leaves, while MP-4 had the fewest leaves. The variation coefficient showed that the plant height of the *M. polymorpha* accessions changed the most (43.15%), followed by branch number (33.42%), and stem-leaf ratio and stem diameter changed the least (9.60% and 4.84%, respectively). Additionally, as the correlation analysis in Table 3 shows, leaf length and leaf width were significantly positively correlated, and leaf length and leaf width were both significantly positively correlated with plant height. Branch number, stem diameter, and stem-leaf ratio were positively correlated with each other, indicating that the *M. polymorpha* accessions with thicker stem had greater branch numbers.

**Table 2:** Statistics for six phenotypic characters recorded for 10 *M. polymorpha* accessions.

Code	Leaf length (cm)	Leaf width (cm)	Plant height (cm)	Branch number (*n*)	Stem diameter (cm)	Stem-leaf ratio
MP-2	2.01 ± 0.19	1.79 ± 0.14	22.20 ± 2.55	1.90 ± 0.08	1.84 ± 0.12	1.55 ± 0.32
MP-3	1.48 ± 0.10	1.51 ± 0.10	4.05 ± 0.59	4.23 ± 0.90	2.03 ± 0.04	1.73 ± 0.44
MP-4	1.38 ± 0.21	1.45 ± 0.12	3.96 ± 0.65	3.80 ± 0.28	1.97 ± 0.19	1.78 ± 0.22
MP-5	2.17 ± 0.04	2.04 ± 0.08	20.47 ± 1.21	2.33 ± 0.24	1.72 ± 0.14	1.62 ± 0.12
MP-6	2.40 ± 0.37	2.23 ± 0.34	22.22 ± 4.63	1.87 ± 0.49	1.86 ± 0.13	1.34 ± 0.21
MP-7	2.27 ± 0.23	2.09 ± 0.22	23.89 ± 6.49	2.03 ± 0.40	1.89 ± 0.06	1.53 ± 0.15
MP-8	2.14 ± 0.18	1.95 ± 0.10	20.49 ± 2.73	2.13 ± 0.26	1.79 ± 0.14	1.48 ± 0.06
MP-9	2.24 ± 0.11	1.96 ± 0.09	20.31 ± 2.60	2.00 ± 0.43	1.89 ± 0.05	1.33 ± 0.07
MP-11	2.32 ± 0.29	2.07 ± 0.17	24.08 ± 4.89	2.13 ± 0.83	1.87 ± 0.13	1.46 ± 0.03
MP-13	1.99 ± 0.09	1.81 ± 0.08	15.11 ± 2.24	3.10 ± 1.06	1.97 ± 0.19	1.59 ± 0.09
The minimum value	1.38	1.45	3.96	1.87	1.72	1.33
The maximum value	2.40	2.23	24.08	4.23	2.03	1.78
The average value	2.04	1.89	17.68	2.55	1.88	1.54
Coefficient of variation	16.98%	13.36%	43.15%	33.42%	4.84%	9.60%

**Table 3:** Correlation coefficients among the phenotypic characters of 10 *M. polymorpha*.

	Leaf length (cm)	Leaf width (cm)	Plant height (cm)	Branch number (*n*)	Stem diameter (cm)	Stem-leaf ratio
Leaf length (cm)	1.000					
Leaf width (cm)	0.977**	1.000				
Plant height (cm)	0.951**	0.892**	1.000			
Branch number (*n*)	−0.339	−0.252	−0.443	1.000		
Stem diameter (cm)	0.046	0.091	−0.061	0.709**	1.000	
Stem-leaf ratio	−0.203	−0.129	−0.221	0.829**	0.677**	1.000

**Note:**

** Significant correlation (*P* < 0.01).

### Cluster analysis of phenotypic traits

**Figure 1:**
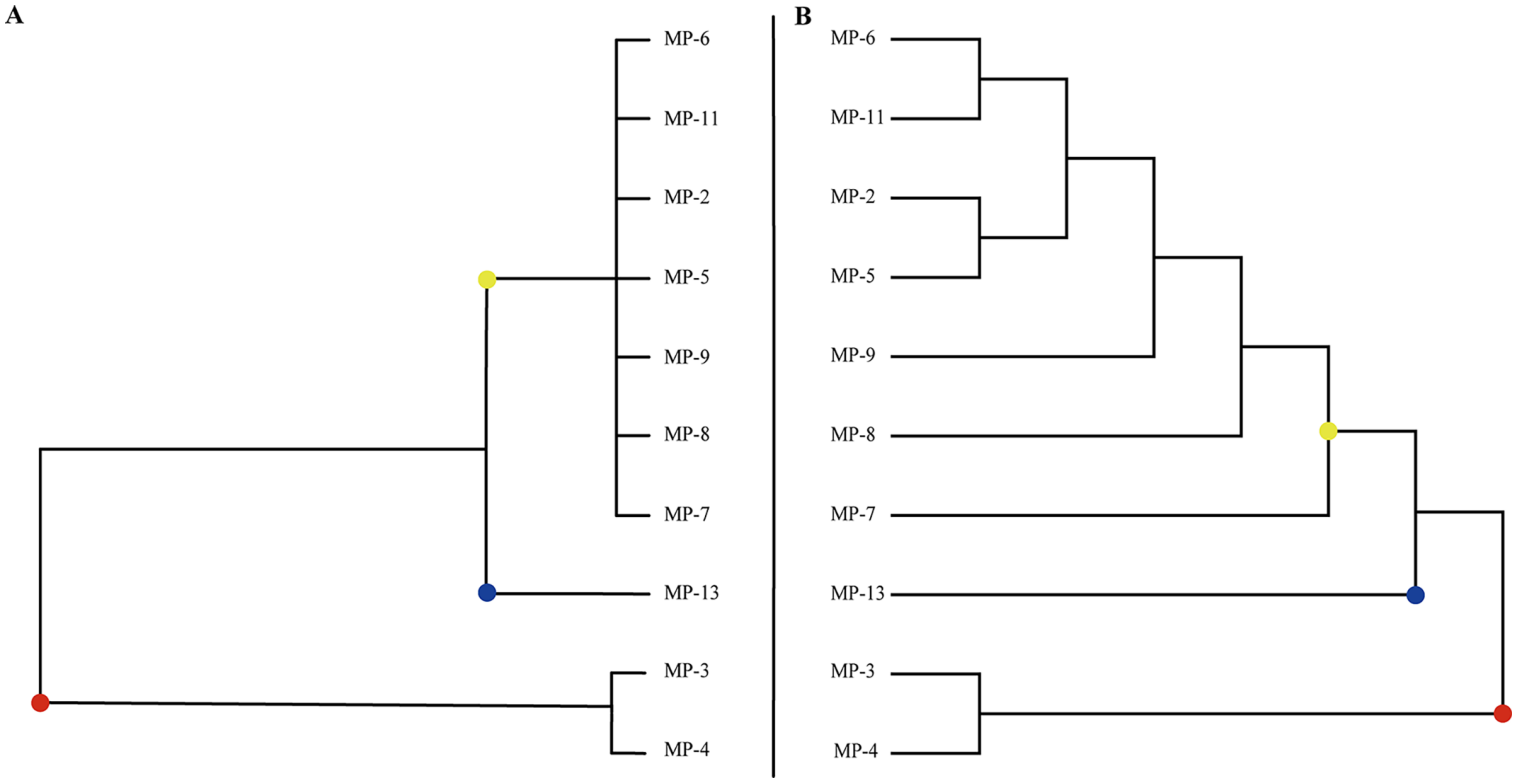
The classification of all *M. polymorpha* accessions. (A) Morphological cluster of total *M. polymorpha* accessions. (B) Phylogenetic tree of 10 *M. polymorpha* accessions reconstructed by maximum likelihood estimation method.

Clustering 10 *M. polymorpha* accessions based on their phenotypic traits (Fig. 1A) revealed three groups and a high value of genetic diversity among various accessions. The first group contained 70% of the studied accessions from all geographical regions, including MP-5, MP-6, MP-7, MP-8, MP-9, and MP-11. The second group was comprised of 10% of the accessions assessed. The third group was made up of 20% of the genotypes, including MP-3 and MP-4. MP-13 was assigned to a separate cluster due to its approximate average value for all phenotypic traits among the evaluated genotypes.

### SLAF library validity and SLAF-seq

Since the genome of *M. polymorpha* was not published at the time of this study, we determined that the “*Rsa*I + *Hae*III” double-enzyme digestion scheme was the best enzyme digestion scheme for constructing the *M. polymorpha* SLAF tag library based on the prediction results of electronic digestion of the *M. truncatula* genome by Biomarker Technologies Corporation (Beijing, China). The selected size for this method was 314–414 bp using the Blue Pippin system. We predicted that there were 117,528 SLAF tags distributed in the genome with 6.19% located in repetitive regions. To further evaluate the efficiency of the restriction enzyme digestion scheme, *Arabidopsis thaliana* was selected as an example. Through the detection of the remaining restriction enzyme restriction sites in *Arabidopsis* read inserts, we found that the ratio of Arabidopsis paired-ended reads mapped to their reference genome was 83.41%, and the enzyme digestion efficiency was 97.79%. This indicates that the double-enzyme digestion scheme should be effective for the SLAF library construction of *M. polymorpha*.

SLAF sequencing of the 10 *M. polymorpha* accessions generated 6.26 Gb clean reads, with a Q30 percentage of 84.97% and GC content of 38.29% (Table S1). Finally, a total of 87,207 SLAF tags were identified, and a single accession contained 79,008 to 85,727 tags (mean 82,336 tags). The read numbers for SLAF tags ranged from 598,481 to 1,869,872 (mean 944,305), and the sequencing coverage ranged from 7.40-fold to 21.88-fold (mean 11.37-fold). Of the identified 87,207 SLAF markers, 25,616 (29.37%) were polymorphic and 70.53% were non-polymorphic, and only 0.1% were repetitive (Table S2). The integrity values of SLAFs from *M. polymorpha* accessions were all above 90% (Table S3). The high quality of these data promised the reliability of the subsequent analyses.

### Development of SNP and SSR markers

Only polymorphic SLAF markers were further selected as high quality SNPs. PCR duplicates were not removed after alignment since the removal would result in many sites in the results not covered by reads, or very low coverage. Moreover, SLAF sequencing does not sequence the entire genome, but sequences the digested fragments according to the restriction sites. Therefore, data filtering strategies used the following parameters: QD >2, MQ >40.0, MQRankSum >−12.5, ReadPosRankSum >−8. After aligning the fragments to the *M. polymorpha* reference genome, a total of 52,237 SNPs with integrity > 90% and minor allele frequency (MAF) >0.05 were identified in this study (Table S4). The MAF distribution spectrum is listed in Table S5. These SNP variants were mostly distributed on pseudochromosome 3 (22.21%), least on pseudochromosome 7 (7%), and nearly evenly distributed on the five other pseudochromosomes (~14.16% for each pseudochromosome).

In order to provide a theoretical basis for the development of SSR molecular markers in *M. polymorpha*, the distribution characteristics of the SSR sequence were analyzed using MISA software. Results showed that 195,753 complete SSR sequences were screened, with relative densities of 428/Mb. The total length of SSR sequences was 3,611,698 bp and accounted for 0.79% of the total genome sequence length. Among the one to six different nucleotide repeats units, the single nucleotide repeat units were the most, followed by dinucleotide, trinucleotide, tetranucleotide, pentanucleotide, and hexanucleotide repeat units. SSR repeat unit types in the *M. polymorpha* genome were abundant and had great potential for developing polymorphic markers and in the genetic diversity and molecular marker-assisted breeding of *M. polymorpha*.

### Genetic diversity of *M. polymorpha* based on SNP markers and agronomic traits

**Figure 2:**
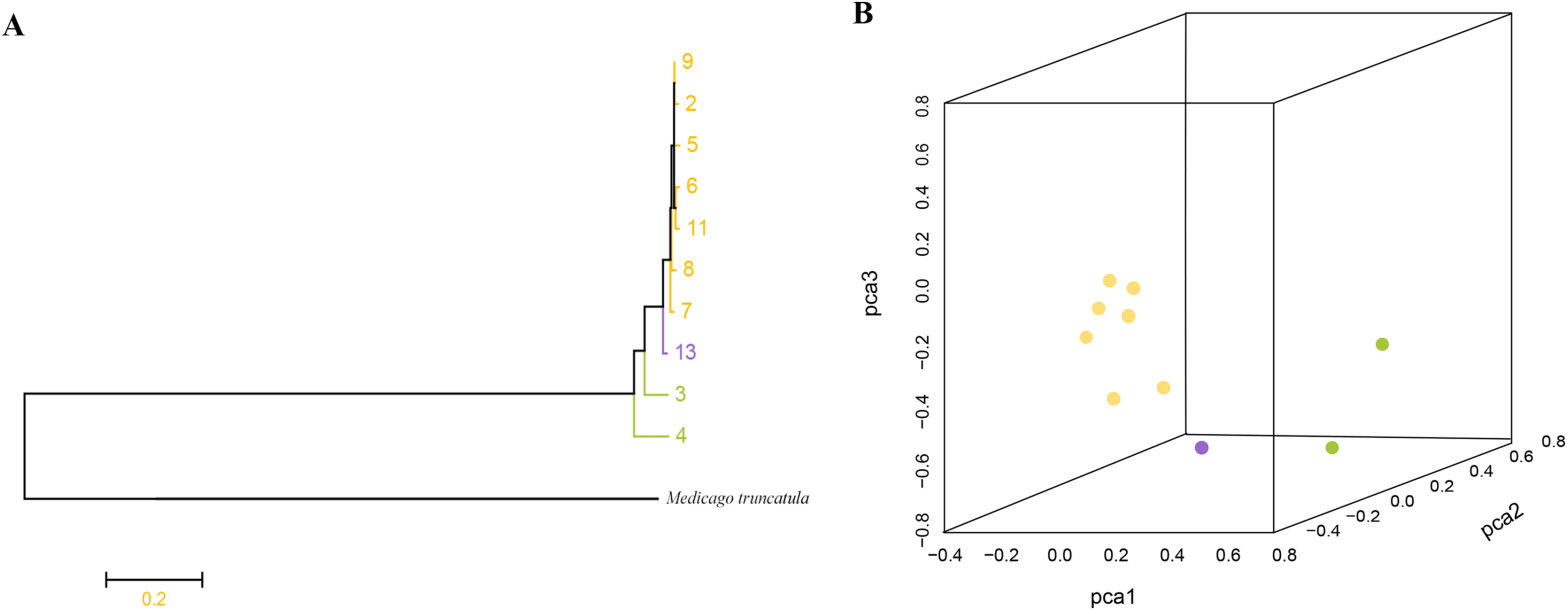
(A) Root evolutionary tree of total *M. polymorpha* accessions with *M. truncatula* as outgroup. (B) Principal components analysis.

Phylogenetic analysis based on high-quality SNPs also showed that all *M. polymorpha* accessions were divided into three clades (Fig. 1B), consistent with morphological clustering (Fig. 1A). The first clade contained MP-2, MP-5, MP-6, MP-7, MP-8, MP-9, and MP-11. The second clade included only MP-13, and the third clade had two accessions, MP-3 and MP-4. When *M. truncatula* was used as the outgroup, the rooted evolutionary trees (Fig. 2A) suggested that MP-3 and MP-4 were the most related to the putative ancestral species of the *M. polymorpha* population. Furthermore, PCA was performed and the first, second, and third principal components explained 14.88%, 12.75%, and 11.92% of the genetic diversity, respectively. The first principal component roughly separated the three groups (Fig. 2B). For the three clusters (Fig. 1A), the He, Nei, I, and PIC values of group I were the highest, followed by group III, and then group II (Table 4). These results indicated that group I possessed the highest genetic diversity, while group II had the lowest genetic diversity.

**Table 4:** Genetic diversity parameters of the different populations identified in 10 *M. polymorpha* accessions based on SNPs from SLAF-seq.

Subpopulation	Ho	He	Nei	I	PIC	MAF
Group I	0.29	0.39	0.48	0.58	0.37	0.31
Group II	0.21	0.30	0.32	0.47	0.25	0.22
Group III	0.25	0.33	0.37	0.50	0.27	0.24

**Note:**

Ho, observed heterozygosity; He, expected heterozygosity; Nei, Nei diversity index; I, Shannon’s information index; PIC, polymorphic information content; MAF, minor allele frequency.

## Discussion

*M. polymorpha* is native to the areas surrounding the Mediterranean Sea and has been naturalized in most parts of the world. Molecular marker development and diversity evaluation of *M. polymorpha* accessions collected from different regions in China can provide useful information concerning sustainable conservation and the utilization of genetic diversity. However, previous genomic studies on *M. polymorpha* have been limited compared to applicable molecular markers in other major crops. Genomic data published by Cui et al. (2021) provided researchers novel insight into *M. polymorpha* molecular marker development. In this study, we used 10 *M. polymorpha* accessions collected from different regions of China to sequence genome-wide distributed SLAFs for polymorphism detection and genotyping for the first time.

In recent years, SLAF-seq technology has been widely used for high-throughput SNP marker development, QTL mapping, high-density genetic map construction, the genetic divergence analysis, and genome-wide association analyses of important agronomic traits in major crops. Wang et al. (2019) used SLAF-seq to construct a genetic map including 7,033 SNP loci that covered 3,353.15 cM with an average distance between consecutive markers of 0.48 cM, and total 13 stable QTL associated with six cottonseed quality traits were detected, Wei et al. (2020) constructed a SNP-based genetic map of Eggplant based on large-scale SNP markers discovered by SLAF-seq technology and QTL Analysis. Huang et al. (2021) identified 60,495 polymorphic SNPs in a subset upland cotton MAGIC population containing 372 lines using SLAF-seq technology, and subsequently successfully detected eight genes with known functions associated with important agronomic traits. Li et al. (2021) investigated the genetic diversity and population structure of weedy and cultivated broomcorn millets by using the SLAF-seq technology. Wang et al. (2022) identified a candidate region of pmHYM in the chromosome 7BL of wheat landrace cultivar Hongyoumai using SLAF and BSR-seq methods. Likewise, Xue et al. (2022) constructed a high-density genetic map containing 9,980 SLAF markers using SLAF-seq, and identified two QTLs related to palmitic acid content in soybean. In summary, previous research has indicated that SLAF-seq technology is a highly efficient method used in molecular marker development and diversity evaluation analysis.

In our study, we identified a total of 25,616 polymorphic SLAF tags containing 52,237 high-consistency SNPs with MAF >0.05 and integrity >0.9. The identified SNPs were mostly distributed on pseudochromosome 3 (22.21%), least on pseudochromosome 7 (7%), and nearly evenly distributed on the other pseudochromosomes of *M. polymorpha*. Moreover, 195,753 complete SSR sequences were screened, with relative densities of 428/Mb. SSR repeat unit types in the *M. polymorpha* genome were abundant and showed great potential for developing polymorphic markers, as well as important application value in the genetic diversity and molecular marker-assisted breeding of *M. polymorpha*.

In order to evaluate the genetic diversity of *M. polymorpha*, a variety of molecular markers have been developed, including isozyme, random amplified polymorphic DNA (RAPD), and SSR (Paredes et al., 2002; Chu et al., 2010; Emami-Tabatabaei et al., 2021). However, these studies had the shortcomings of having a limited number of markers and being time-consuming and laborious. In this study, we developed more than 50,000 high-quality SNPs of *M. polymorpha* using SLAF-seq for the first time. Our results showed that *M. polymorpha* exhibited a considerable level of nucleotide diversity. However, *M. polymorpha* is a niche species, and there has not been a standardized collection of germplasm resources in most areas of China. Due to this limitation, we collected only 10 *M. polymorpha* accessions in China under the existing conditions. Nevertheless, previous studies have shown that small samples tend to produce an excess number of intermediate frequency alleles than large samples (Ramírez-Soriano & Nielsen, 2009; Tenaillon et al., 2001), and analyses of genetic diversity based on small populations can still be important (Nayanakantha, Singh & Gupta, 2010; Zhang et al., 2019), especially for *M. polymorpha*.

The evaluation of genetic diversity with both agronomic traits and molecular markers can be useful to analyze genetic diversity within species with more precision, as well as in phylogenetic studies (Ambreen et al., 2018). Co-analysis of agronomic traits and molecular data have extensively been used for genetic diversity analysis in various crops such as *Triticum urartur* (Wang et al., 2017) and safflower (Golkar, Arzani & Rezaei, 2011), but it has not been studied in *M. polymorpha*. In this study, we analyzed the phenotypic characteristics of *M. polymorpha* materials and found that there were great differences in the phenotypic traits across different *M. polymorpha* accessions. Cluster analysis of all 10 *M. polymorpha* accessions based on their phenotypic traits indicated that these accessions could be grouped into three groups, which was basically consistent with their phenotypic traits. Moreover, phylogenetic and PCA analyses based on SNP markers also showed that all *M. polymorpha* accessions were divided into three clades, which was consistent with morphological clustering results.

Large numbers of SNP markers discovered by SLAF-seq technology in *M. polymorpha* germplasm collected from different regions of China can provide an important breeding resource and lay the foundation for future genetic analysis. Previous studies have shown that using a combination of SNP markers and agronomic traits is an effective method for evaluating genetic diversity (Golkar & Nourbakhsh, 2019), which was also confirmed in our study.

## Conclusion

In this study, we applied SLAF sequencing technologies to develop molecular markers and explore the genetic diversity of *M. polymorpha* germplasm collected from different regions in China. More than 50,000 high-confidence SNP markers in a panel of 10 *M. polymorpha* accessions were first developed. These SNP markers were evenly distributed in each chromosome, with the most on pseudochromosome 3 (22.2%) and the least on pseudochromosome 7 (7.0%). Cluster analysis of all *M. polymorpha* accessions based on SNP markers (phylogenetic analysis and PCA analysis) and phenotypic traits both suggested that they could be divided into three groups. Subsequent genetic diversity analysis for these 10 accessions also validated the effectiveness of the molecular markers of *M. polymorpha* germplasm in China. Additionally, we performed SSR mining analysis to identify polymorphic SSR motifs, which helped to provide valuable candidate markers for the subsequent breeding of *M. polymorpha*. Our study will provide important information about the molecular markers of *M. polymorpha* and will be useful for further research on *M. polymorpha* resource utilization and marker-assisted breeding.

## Supplemental Information

10.7717/peerj.14698/supp-1 Supplemental Information 1 Supplemental Tables.

## Additional Information and Declarations

### Data Availability

The following information was supplied regarding data availability: The data is available at GenBank: PRJNA339643.
